# Hepatitis E virus-associated neurological injury and neurotropic cellular mechanisms

**DOI:** 10.3389/fcimb.2026.1810452

**Published:** 2026-04-22

**Authors:** Zixuan Guo, Yuewei Tian, Minheng Cheng, Wenjiao Yin, Yaxin Sun, Yubing Ou, Xiujuan Peng, Tianlong Liu, Ruiping She, Yanxin Hu, Jijing Tian

**Affiliations:** 1 Laboratory of Animal Pathology and Public Health, State Key Laboratory of Veterinary Public Health and Safety, College of Veterinary Medicine, China Agricultural University, Beijing, China; 2 Development Center of Science and Technology, Beijing Municipal Bureau of Agriculture and Rural Affairs, Beijing, China; 3 National Institute for Viral Disease Control and Prevention, Chinese Center for Disease Control and Prevention, NHC Key Laboratory of Medical Virology and Viral Diseases, Beijing, China

**Keywords:** central nervous system, hepatitis E virus, immune-mediated injury, neurological manifestations, peripheral neuropathy

## Abstract

Hepatitis E virus (HEV) is increasingly recognized as a cause of neurological disease beyond its hepatic manifestations. Neurological complications are the most frequently reported extrahepatic presentations and include both peripheral nervous system disorders, such as Guillain–Barré syndrome (GBS) and neuralgic amyotrophy (NA), and central nervous system (CNS) involvement, including encephalitis and myelitis, often in the absence of overt hepatitis. This review summarizes the clinical spectrum of HEV-associated neurological disease and integrates evidence from human studies and experimental models. Current evidence supports multifactorial pathogenesis, with direct viral neuroinvasion of the CNS and immune-mediated mechanisms predominating in peripheral neuropathies. Experimental *in vivo* and *in vitro* systems demonstrate that HEV can cross the blood–brain barrier (BBB) and replicate within neural tissues, providing biological plausibility for CNS involvement. By synthesizing clinical and experimental findings, this review highlights the dual pathogenic pathways underlying HEV-associated neurological injury and outlines key unresolved questions relevant to diagnosis, pathogenesis, and clinical management.

## 1 Introduction

Hepatitis E virus (HEV) is one of the leading causes of enterically transmitted viral hepatitis worldwide. It has been estimated that approximately 939 million people have ever experienced HEV infection (Li et al., 2020). In addition to acute and chronic liver disease, HEV is increasingly recognized as a cause of extrahepatic disorders, with neurological syndromes being the most frequently reported. Neurological complications are reported in up to 30% in some cohorts of diagnosed infections, making them the most common extrahepatic manifestation (Wiesenfarth et al., 2024). These conditions range from peripheral nerve disorders such as Guillain–Barré syndrome (GBS) and neuralgic amyotrophy (NA) to central nervous system (CNS) involvement including encephalitis and myelitis, and they often occur in patients with minimal or no hepatic symptoms (Jha et al., 2021). This review summarizes the clinical spectrum of HEV-associated neurological disease and integrates findings from human studies and experimental models to clarify current concepts of pathogenesis, including immune-mediated mechanisms and direct viral neuroinvasion.

## 2 HEV genotypes, host range, and zoonotic transmission

**Figure 1:**
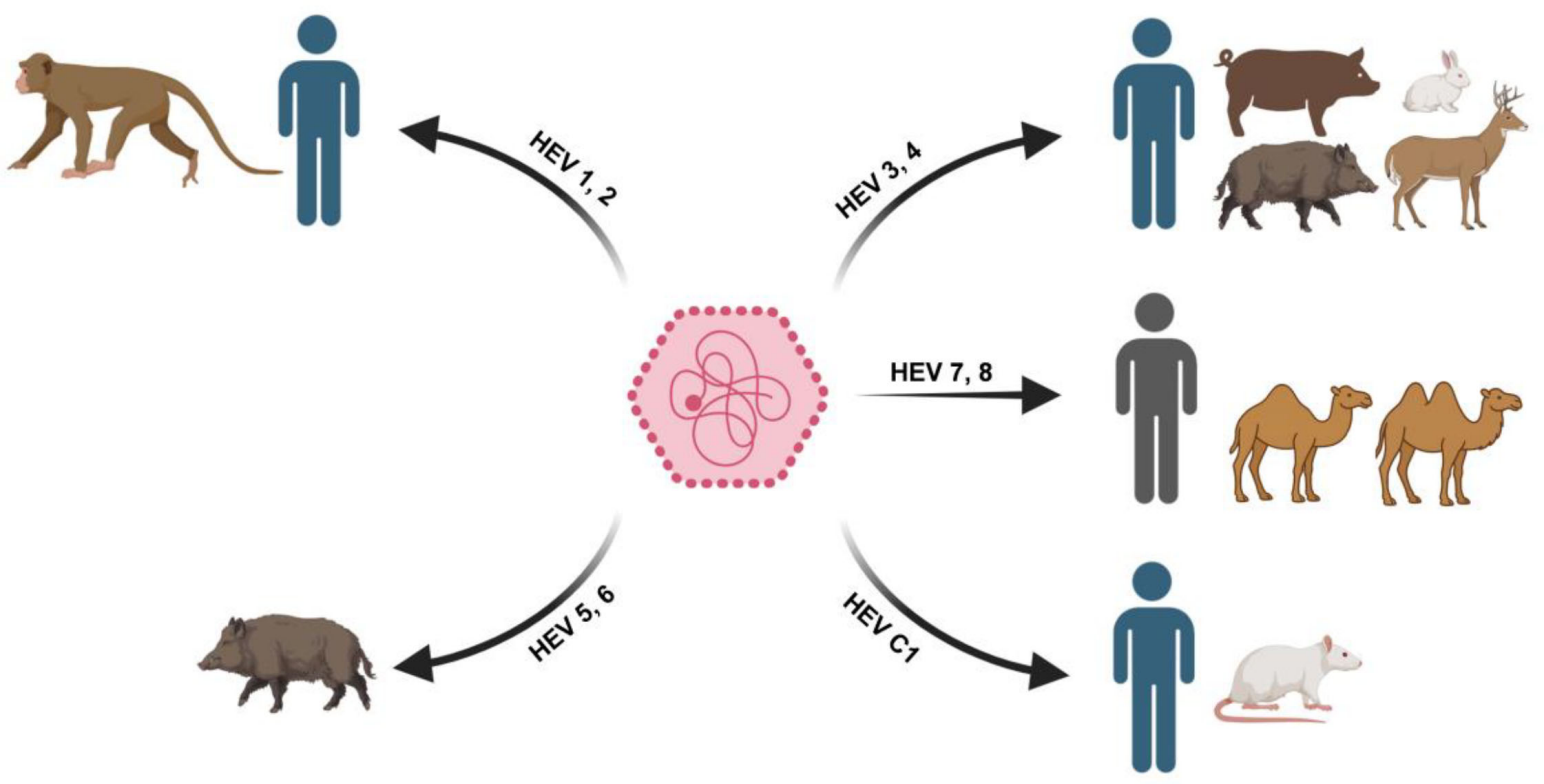
Genotypes, host range and transmission of HEV. Human-restricted genotypes HEV-1 and HEV-2 circulate exclusively in humans and are transmitted primarily via waterborne fecal-oral routes. Zoonotic genotypes HEV-3 and HEV-4 are maintained in animal reservoirs, including pigs, wild boar, and deer, and infect humans mainly through foodborne exposure or direct contact. Additional animal-associated genotypes include HEV-5 and HEV-6 in wild boar, HEV-7 and HEV-8 in camels, and the rat-associated hepevirus HEV-C1 (*Rocahepevirus ratti*), which can spill over to humans. Together, the figure illustrates the One Health nature of HEV epidemiology and the role of cross-species transmission in human infection. Some schematic elements in this figure were created with BioRender.com.

HEV is a quasi-enveloped, positive-sense, single-stranded RNA virus classified within the family *Hepeviridae*, which comprises several genera infecting mammals, birds, and fish. Human-pathogenic HEV strains correspond to the species *Paslahepevirus balayani* within the subfamily *Orthohepevirinae* and include at least eight genotypes (HEV-1 to HEV-8), several of which differ in host range and predominant transmission patterns (Purdy et al., 2022) (**Figure 1**). Genotypes 1 and 2 are restricted to humans and are primarily transmitted via waterborne fecal-oral routes, accounting for large outbreaks in developing regions (Wang and Meng, 2021). In contrast, genotypes 3 and 4 are zoonotic, with domestic pigs, wild boar, and deer serving as major reservoirs, and cause sporadic infections in industrialized countries, frequently associated with the consumption of undercooked meat or animal-derived products (Dalton and Izopet, 2018). Additional genotypes include HEV-5 and HEV-6, which circulate mainly in wild boar, and HEV-7 and HEV-8, which are associated with camels. Notably, HEV-7 has been shown to infect humans, as demonstrated by a chronic infection following the consumption of camel meat (Velavan et al., 2021).

Beyond the eight genotypes of *Paslahepevirus balayani*, other members of the family *Hepeviridae* further expand the zoonotic landscape of hepatitis E-like infections. In particular, *Rocahepevirus ratti* (formerly *Orthohepevirus C*), a divergent rat-associated hepevirus, has been identified as a cause of clinically significant hepatitis in humans, predominantly in immunosuppressed individuals. Importantly, extrahepatic involvement has been documented, including a fatal case of meningoencephalitis in which HEV-C1 RNA was detected in the cerebrospinal fluid (Sridhar et al., 2021). Together, these findings underscore that hepatitis E-related disease extends beyond classical HEV genotypes and highlight the central role of animal reservoirs and cross-species transmission in shaping the epidemiology and emergence of hepevirus infections in humans.

## 3 Neurological manifestations of HEV infection in humans

In addition to hepatic disease, HEV can cause extrahepatic manifestations, with neurological complications representing the most frequently reported and clinically relevant entities. Since the first description of Guillain–Barré syndrome (GBS) following acute HEV infection in 2000 (Sood et al., 2000), a broad spectrum of peripheral and CNS disorders has been documented worldwide, including neuralgic amyotrophy (NA), encephalitis, myelitis, and Bell’s palsy (Fousekis et al., 2020). Reported frequencies of neurological involvement range from approximately 5% to 30% across cohorts, with higher estimates observed in studies employing systematic neurological evaluation rather than passive case ascertainment (Ripellino et al., 2020; Jha et al., 2021). Importantly, many patients are anicteric and have normal or only mildly abnormal liver function tests, which may delay diagnosis (Dalton et al., 2017); therefore, HEV should be considered in the differential diagnosis of otherwise unexplained acute neurological syndromes, even in the absence of overt hepatitis (Iza et al., 2025).

### 3.1 Peripheral nervous system involvement: GBS and NA

GBS is an acute immune-mediated polyneuropathy and represents one of the most frequently reported neurological complications of HEV infection. Multiple case-control studies from Asia and Europe have reported an association between acute HEV infection and GBS, although results are not fully consistent across cohorts, suggesting potential geographic or subgroup heterogeneity (van den Berg et al., 2014; Fukae et al., 2016; Lhomme et al., 2021; Ripellino et al., 2024). Clinically, HEV-associated GBS typically presents as acute flaccid paralysis occurring shortly after or during HEV infection and often shows demyelinating features on nerve conduction studies; cranial nerve involvement and Miller Fisher syndrome variants have also been described (Fukae et al., 2016; Zheng et al., 2018; Zhou et al., 2023). The detection of anti-ganglioside antibodies, including GM1 and GM2, in a subset of patients supports an immune-mediated pathogenesis (Loly et al., 2009; Zheng et al., 2018; Liu and Ma, 2020). Management follows standard GBS protocols, including intravenous immunoglobulin or plasma exchange with supportive care (Leonhard et al., 2019). Ribavirin may be considered in selected patients with ongoing HEV viremia, although convincing evidence for improved neurological outcomes remains lacking (European Association for the Study of the, 2018; Lhomme et al., 2021).

NA is characterized by abrupt, severe shoulder or arm pain followed by patchy brachial plexus weakness and atrophy (Lhomme et al., 2021). HEV has emerged as a relevant infectious trigger, particularly in regions where genotype 3 predominates, and several cohorts indicate that a subset of otherwise idiopathic NA cases show evidence of recent HEV infection at symptom onset (van Eijk et al., 2014; Fritz et al., 2018; Bannasch et al., 2021; Ripellino et al., 2024). A recent multicenter, prospective, matched case-control study demonstrated a significant association between acute HEV infection and NA, whereas no association was observed for GBS or Bell’s palsy in the same analysis (Ripellino et al., 2024). HEV-associated NA frequently displays a distinctive phenotype with bilateral involvement and extra-brachial nerve manifestations, including phrenic nerve paresis, supporting targeted HEV testing in patients with bilateral or extensive disease (van Eijk et al., 2017; Leonhard et al., 2022; Mackrill et al., 2023). There is no proven disease-modifying therapy for NA; management focuses on analgesia and rehabilitation, with short courses of corticosteroids sometimes used for severe pain. Identification of active HEV infection may prompt consideration of antiviral therapy, although ribavirin has not been shown to consistently improve neurological outcomes (van Eijk et al., 2017; Fraga et al., 2018; Bannasch et al., 2021)

### 3.2 CNS involvement: encephalitis and myelitis

HEV can involve the CNS and present as encephalitis or meningoencephalitis with seizures, altered mental status, or focal neurological deficits (Jha et al., 2021; Lhomme et al., 2021). Cerebrospinal fluid findings are heterogeneous, and HEV RNA is inconsistently detectable; therefore, diagnosis should not rely on CSF PCR alone but instead integrate serology, plasma or stool nucleic-acid testing, and clinicoradiological correlation (Murkey et al., 2017; Abravanel et al., 2021; Jha et al., 2021; Hafkesbrink et al., 2025). Metagenomic next-generation sequencing has further expanded diagnostic capability and has identified HEV directly from CSF in selected cases, highlighting its value in diagnostically unresolved meningoencephalitis (Murkey et al., 2017; Benoit et al., 2024). In immunosuppressed hosts, zoonotic HEV lineages can also invade the CNS, as illustrated by meningoencephalitis caused by rat HEV with detectable viral RNA in CSF (Sridhar et al., 2021). Management is primarily supportive; ribavirin may be considered when active viral replication is documented, although evidence for neurological benefit is limited, and immunomodulatory therapy such as corticosteroids may be used on a case-by-case basis when an immune-mediated component is suspected (European Association for the Study of the, 2018; Hafkesbrink et al., 2025).

Acute transverse myelitis (ATM) is an established but uncommon CNS manifestation of HEV infection, typically presenting with acute motor or sensory deficits and a discrete spinal sensory level (Beh et al., 2013; Jha et al., 2021). Evidence is largely derived from case reports and small series in both adults and children, including instances with detection of HEV RNA in serum and cerebrospinal fluid, supporting a causal association (Mandal and Chopra, 2006; Sarkar et al., 2015; Rahmig et al., 2020). More recent observations in immunocompromised patients describe chronic HEV infection with recurrent myelopathy and spinal cord atrophy, indicating that sustained CNS involvement and persistent viral activity may occur in selected hosts (Ritter et al., 2024). Collectively, available data support a multifactorial pathogenesis involving both immune-mediated injury and direct neuroinvasion, consistent with evidence of CNS compartmentalization of HEV replication (den Drijver et al., 2021; Jha et al., 2021; Lhomme et al., 2021). Management generally follows standard protocols for ATM of other etiologies, including high-dose corticosteroids and early rehabilitation; ribavirin may be considered on a case-by-case basis when active viral replication is documented, although neurological benefit remains unproven (European Association for the Study of the, 2018; Rahmig et al., 2020; den Drijver et al., 2021).

### 3.3 Other neurological complications

Beyond these major syndromes, HEV has been associated with additional, less frequent presentations, including mononeuritis multiplex, small-fiber neuropathy with neuropathic pain, cranial neuropathies such as facial nerve palsy, and occasional muscle involvement ranging from focal myositis to inflammatory myopathy with marked creatine kinase elevation (Angeli and Gines, 2012; Del Bello et al., 2012; Perrin et al., 2015; Mengel et al., 2016; Pischke et al., 2017; Abravanel et al., 2018; Jha et al., 2021; Lhomme et al., 2021). Rare temporal associations with myasthenia gravis and mixed peripheral-central syndromes such as meningoradiculitis or ADEM-like presentations have also been described, although causality is uncertain and evidence is largely limited to case reports and small series (Belbezier et al., 2014; Wang et al., 2018a; Rahmig et al., 2020). Overall, clinicians should consider HEV in otherwise unexplained peripheral neuropathies or CNS inflammation, even when liver function tests are normal or only mildly abnormal (Dalton et al., 2017; Jha et al., 2021).

## 4 *In vivo* and *in vitro* models for HEV-induced neurological injury

**Figure 2:**
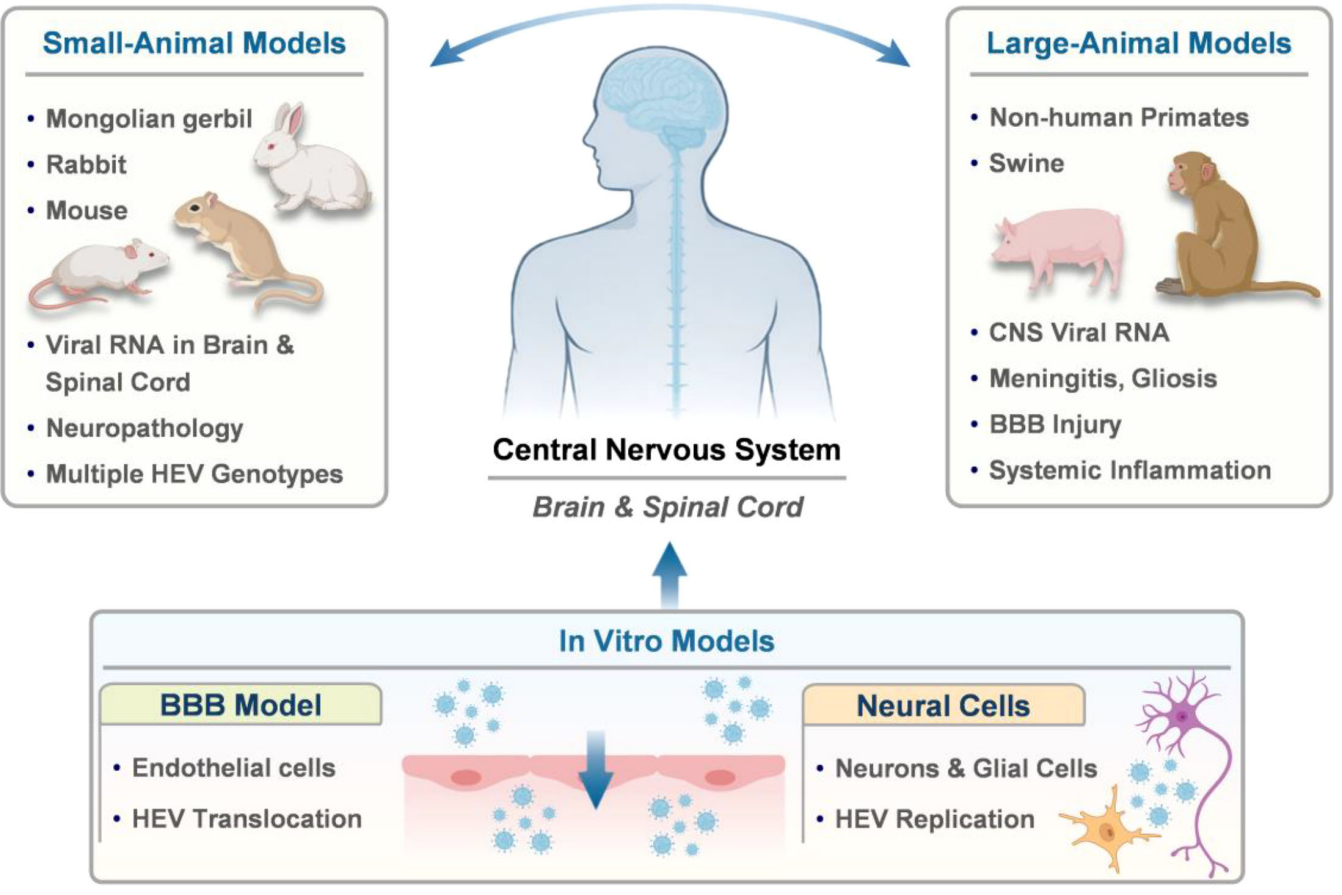
Overview of *in vivo* and *in vitro* experimental models used to study HEV-induced neurological injury. Across small-animal models, large-animal models, and cell-based systems, HEV has been detected in brain and spinal tissues and shown to replicate in neural and endothelial cells, providing convergent evidence for HEV neurotropism. While individual models vary in pathological features, together they support the capacity of HEV to access the CNS across species and experimental platforms. Some schematic elements in this figure were created with BioRender.com.

Animal and cell-based models have been instrumental in establishing the neurotropic potential of HEV. Across diverse experimental systems, HEV has been shown to disseminate beyond the liver, reach the CNS via the circulation, and replicate within brain and spinal tissues, producing neuropathological changes that parallel those reported in human infection. However, current models vary in their ability to reproduce the full spectrum of neurological disease, particularly immune-mediated syndromes, underscoring the need for careful model selection when interpreting experimental findings (**Figure 2**).

Among small-animal models, the Mongolian gerbil has emerged as a robust system for studying HEV neuroinfection. Gerbils are susceptible to multiple HEV subgenotypes, and experimental infection consistently results in the detection of both positive- and negative-strand viral RNA in the brain and spinal cord over a broad post-inoculation window, providing direct evidence of active CNS replication (Li et al., 2009; Shi et al., 2016; Zhang et al., 2022; Liu et al., 2024). Neuropathological analyses reveal marked ultrastructural abnormalities, including endothelial injury, basement membrane thickening, mitochondrial degeneration, and perivascular inflammatory infiltration, indicating blood–brain barrier (BBB) involvement and neural tissue damage (Shi et al., 2016; Tian et al., 2019a, 2019). The sensitivity of this model to pegylated interferon-α2a and ribavirin further supports its utility for evaluating antiviral interventions in the context of HEV neuroinfection (Xu et al., 2022).

Experimental infection of rabbits provides complementary evidence for CNS involvement across species. Rabbits inoculated with swine-derived HEV-4 isolates develop CNS infection characterized by replicative viral RNA in the brain and spinal cord and localization of ORF2 capsid antigen in neural and perivascular regions. The associated histopathological changes, including neuronal injury and inflammatory infiltrates, closely resemble those observed in gerbils, reinforcing the reproducibility of HEV-associated neuropathology in small mammals (Han et al., 2014; Tian et al., 2019a). Consistent findings have also been reported in mice and non-human primates, in which viral RNA and ORF2 protein are detectable in brain tissue during both acute and prolonged infection, supporting the capacity of HEV to access and persist within the CNS across mammalian hosts (Zhou et al., 2017).

Large-animal models further extend these observations under physiologically relevant conditions. In specific-pathogen-free pigs, both quasi-enveloped and non-enveloped genotype 3 HEV strains have been detected in brain and spinal cord tissues following experimental infection, accompanied by histological changes such as meningitis, perivascular inflammation, and gliosis (Tian et al., 2022). Animals with CNS involvement also exhibit enhanced systemic inflammatory responses, highlighting the association between viral dissemination and host inflammatory activation in this model.

*In vitro* systems provide complementary cellular platforms to support observations from animal studies. Human brain microvascular endothelial cells are permissive to HEV infection, and both quasi-enveloped and non-enveloped virions are capable of traversing an *in vitro* BBB model (Tian et al., 2019a; Bannasch et al., 2021). In parallel, multiple human neuronal and glial cell lines, including the oligodendrocyte-derived MO3.13 line, support productive HEV replication, and primary human induced neurons are directly susceptible to infection, providing physiologically relevant *in vitro* correlates of HEV neurotropism (Drave et al., 2016; Fu et al., 2019; Jagst et al., 2024).

Recent advances in human neuron-based *in vitro* systems have provided strong evidence for HEV neurotropism. In human iPSC-derived induced primary neurons (iPNs), HEV-3 efficiently infects mature β-III-tubulin-positive neurons, with preferential susceptibility of neurite-bearing cells and intracellular accumulation of ORF2 capsid protein in perinuclear regions and neuronal processes. Viral replication increases with neuronal maturation and is not effectively suppressed by interferon-α, underscoring the limited intrinsic antiviral responses of neurons and the utility of iPNs for modeling direct HEV-neuron interactions (Jagst et al., 2024). Extending these findings to a higher level of tissue organization, human iPSC-derived brain organoids (hBOs) demonstrate that HEV-3 can establish sustained, productive infection within complex neural tissue, characterized by persistent viral RNA release, successful serial passage, and widespread ORF2 expression across neuronal and glial populations, with preferential infection of glutamatergic and dopaminergic neurons (Liu et al., 2025). In contrast, HEV-4 fails to achieve productive replication in this system, and HEV-3 infection is efficiently suppressed by ribavirin. Together, iPNs and brain organoids constitute complementary human-relevant platforms that bridge reductionist cell culture models and animal systems for investigating HEV-associated neurological injury.

## 5 Pathogenesis of HEV-induced neurological injury

**Figure 3:**
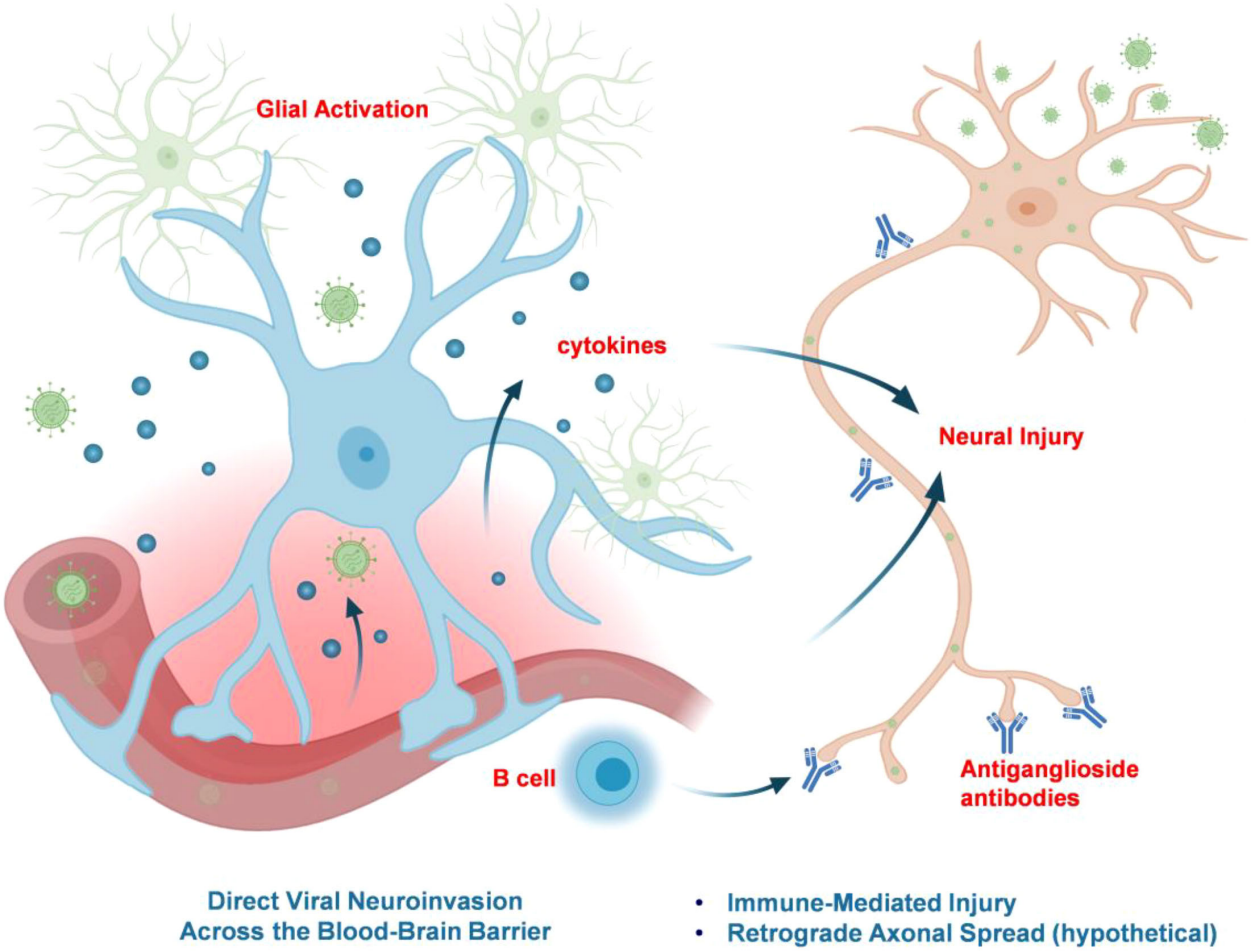
Proposed mechanisms of HEV-associated neuropathogenesis. The diagram illustrates two complementary pathways of HEV-associated neuropathogenesis. Direct viral neuroinvasion (left) involves hematogenous entry across the BBB, infection of neurovascular cells, glial activation, and cytokine release, leading to neuroinflammation and neuronal injury. Immune-mediated injury (right) primarily affects the peripheral nervous system and is driven by systemic immune activation, including B-cell responses and anti-ganglioside antibody production consistent with molecular mimicry. A hypothetical retrograde axonal spread is shown as a possible but unproven route. Together, these mechanisms highlight the multifactorial nature of HEV-induced neurological disease. Some schematic elements in this figure were created with BioRender.com.

The mechanisms by which HEV causes neural damage are an area of active investigation. Current evidence suggests a multifactorial pathogenesis involving both immune-mediated injury and direct viral neurotropism. **Figure 3** summarizes the proposed pathways leading to nervous system injury in HEV infection.

### 5.1 Immune-mediated mechanisms

Growing evidence supports a major role for immune driven injury in HEV associated neurological disease. Epidemiologically, neurological manifestations are reported substantially more often in immunocompetent than immunosuppressed patients, consistent with an immune contribution (Abravanel et al., 2018). Across peripheral nerve syndromes, clinical and immunologic features also align with a post-infectious paradigm. In case control and cohort studies, acute or recent HEV infection has been linked to GBS and related phenotypes, and anti-ganglioside antibodies, including anti-GM1 and anti-GM2, have been detected in a subset of HEV associated cases, supporting molecular mimicry and cross reactive humoral responses against neural glycolipids (Maurissen et al., 2012; Wang et al., 2018b). More broadly, many HEV associated neurological syndromes occur with absent or mild hepatitis and frequently show negative cerebrospinal fluid HEV RNA (Wang et al., 2018b), again favoring immune-mediated injury in many patients, although the relevant autoantigens and the relative contributions of B cell and T cell pathways remain incompletely defined.

Importantly, immune-mediated extrahepatic disease in other organs provides mechanistic precedent that strengthens the plausibility of similar pathways contributing to neural injury. HEV infection has been associated with immune complex related disorders, and a recent kidney transplant study provides direct mechanistic evidence by demonstrating *de novo* immune complex mediated glomerulonephritis that developed alongside progressive glomerular deposition of a non-infectious, genome free, non-glycosylated HEV ORF2 capsid protein, with colocalized IgG and no evidence of productive infection in kidney tissue (Leblond et al., 2024). This work identifies circulating HEV antigen, particularly excess ORF2, as a plausible driver of immune complex deposition and tissue inflammation in the immunocompromised state, offering a concrete framework for how a hepatotropic virus can cause extrahepatic injury without local productive infection. Immune-mediated hematologic injury has also been reported, including immune-mediated thrombotic thrombocytopenic purpura temporally linked to acute HEV infection with ADAMTS13 inhibitor positivity, further supporting that HEV can precipitate antibody mediated pathology beyond the liver (Lv et al., 2023). Together, these extra organ data support a coherent model in which HEV can trigger systemic immune dysregulation and immune complex formation that may contribute to peripheral nerve injury in susceptible hosts even when direct neuroinvasion is not demonstrable.

### 5.2 Direct viral neuroinvasion and neurotropism

Although immune driven mechanisms likely explain many HEV associated neurological syndromes, convergent clinical and experimental data support that HEV can also directly infect the nervous system in at least a subset of patients. A landmark case demonstrated persistent HEV RNA in cerebrospinal fluid for more than one year after clearance from serum and stool, together with intrathecal anti HEV IgG and distinct CSF viral quasispecies, consistent with compartmentalized CNS replication and local viral evolution (den Drijver et al., 2021). CSF specific viral quasispecies have also been reported in patients with neurological manifestations, reinforcing the concept of autonomous CNS infection in selected cases (Abravanel et al., 2021). Importantly, persistent CSF infection may occur even when systemic replication appears controlled, as highlighted by a report of HEV RNA persistence in CSF despite apparently successful ribavirin therapy (Lhomme et al., 2022).

Experimental systems provide mechanistic plausibility for direct neurotropism. *In vivo*, HEV was shown to infect brain microvascular endothelial cells, cross the BBB, and invade the CNS in a controlled model, with viral RNA detected in CNS tissues (Tian et al., 2022). *In vitro*, neuronal and glial derived systems support HEV replication, establishing that neural lineage cells can be permissive (Drave et al., 2016; Zhou et al., 2017). More recently, human induced primary neurons provided a physiologically relevant platform in which HEV established productive infection; neurite-bearing neurons showed higher susceptibility, infected neurons mounted minimal innate antiviral responses compared with hepatocyte models, and infection was associated with neurite shortening, consistent with direct neuronal injury (Jagst et al., 2024). Together, these findings support a complementary pathway to immune-mediated injury in which direct HEV neuroinfection and local replication contribute to neurological disease in a defined subset of patients.

### 5.3 Routes of neuroinvasion: crossing the BBB

Having established that HEV can directly infect neural tissues, a key question is how the virus gains access to the CNS. Current evidence most strongly supports hematogenous entry across the BBB. HEV circulates as both non-enveloped and membrane-associated quasi-enveloped particles, forms that differ in cellular entry pathways and immune shielding (Bruggemann et al., 2025). Using an *in vitro* BBB model and an experimental pig infection system, Tian and colleagues showed that both quasi-enveloped and non-enveloped HEV can traverse the BBB, that TNF-α did not enhance BBB crossing *in vitro*, and that brain microvascular endothelial cells support productive HEV infection (Tian et al., 2022). *In vivo*, HEV RNA was detected in brain and spinal cord tissues of infected pigs and was accompanied by histological lesions and heightened systemic inflammatory responses, supporting BBB-associated neuroinvasion under physiologically relevant conditions (Tian et al., 2022). Experimental studies demonstrate that HEV infects brain microvascular endothelial cells, impairs barrier properties, and reduces the expression of key tight-junction proteins including ZO-1, occludin, and claudin-5 (Tian et al., 2019a, 2022), consistent with barrier dysfunction that could facilitate CNS entry.

An alternative route is retrograde spread along peripheral nerves, a mechanism well established for several neurotropic viruses but not yet demonstrated for HEV. Although this pathway cannot be formally excluded, recent clinical and experimental overviews indicate that available data more consistently support blood-borne dissemination with involvement of the BBB rather than a defined axonal transport mechanism (Luo et al., 2024). Accordingly, peripheral nerve spread should currently be regarded as a theoretical possibility, whereas hematogenous entry across the neurovascular interface is supported by convergent *in vitro* and *in vivo* evidence.

## 6 Knowledge gaps and future directions

Despite growing recognition of HEV associated neurological disease, its true incidence and full clinical spectrum remain underestimated due to inconsistent HEV testing in patients with acute neurological syndromes, particularly in the absence of hepatitis. Immune mediated mechanisms are strongly implicated, yet the relevant neural autoantigens and the relative contributions of humoral and cellular immunity remain undefined, and direct evidence within neural tissues is limited. The recent demonstration of ORF2 driven immune complex disease in the kidney provides a mechanistic precedent (Leblond, 2026), but whether similar processes contribute to peripheral nerve injury has not been directly tested.

For central nervous system involvement, experimental studies support blood-brain barrier crossing and neural permissiveness (Tian et al., 2022), but the frequency of these events in humans and the factors enabling CNS compartmentalization remain unclear. Current models primarily emphasize endothelial infection and barrier dysfunction, while alternative mechanisms of neuroinvasion remain insufficiently explored. In particular, quasi-enveloped HEV is released through an exosome-like pathway and carries extracellular vesicle-associated markers (Nagashima et al., 2017), raising the possibility that vesicle-mediated transport may facilitate immune evasion or CNS entry. However, whether extracellular vesicles directly contribute to blood–brain barrier traversal, transcytosis, or compartmentalized CNS infection has not been experimentally demonstrated. Clinically, neurological recovery does not consistently parallel viral clearance (den Drijver et al., 2021), further underscoring the need for mechanism-informed diagnostic strategies and targeted therapeutic studies. Addressing these gaps will require integrated clinical cohorts and experimental systems that more faithfully recapitulate both immune and neurovascular aspects of HEV infection.

## 7 Conclusion

Hepatitis E virus is increasingly recognized as a clinically relevant cause of neurological disease involving both the peripheral and central nervous systems. Current evidence indicates multifactorial pathogenesis, with immune-mediated mechanisms playing a major role in peripheral neuropathies and direct viral neuroinvasion contributing to central nervous system involvement in a subset of cases. Continued integration of clinical observations with relevant experimental models will be critical to clarify disease mechanisms and to inform more precise diagnostic and therapeutic approaches for HEV-associated neurological disorders.
